# Enhancing the Efficacy, Utility, and Throughput of the Transcription Block Survival Peptide Library Screening Platform

**DOI:** 10.1021/jacsau.5c00628

**Published:** 2025-10-30

**Authors:** Andrew Brennan, T M Simon Tang, Jody M Mason

**Affiliations:** Department of Life Sciences, 1555 University of Bath, Bath BA2 7AY, United Kingdom

**Keywords:** peptide antagonist, protein−protein interactions, transcription factor, ATF2

## Abstract

Genetically encoded peptide library screening is a powerful strategy for discovering inhibitors of protein–protein and protein–DNA interactions. The Transcription Block Survival (TBS) assay enables the *in vivo* selection of peptides that antagonize transcription factor (TF) function by linking the inhibition of DNA binding to *E. coli* survival. However, previous TBS implementations required laborious re-engineering of the mDHFR coding region for each new target, limiting utility. Here, we present an enhanced and streamlined TBS platform that increases throughput, simplifies target switching, and improves selection stringency. By relocating TF DNA-binding sites from within the mDHFR coding sequence into the mDHFR 5′-promoter/untranslated region, we preserve mDHFR folding and function, enabling rapid interchange of TF targets without the need for extensive construct redesign. We validated this system using three distinct TF targets, CREB1, ATF2, and DLX5, and two distinct consensus sites, demonstrating robust transcriptional block upon TF binding and efficient growth rescue upon peptide-mediated antagonism. Importantly, we expand the platform to accommodate full-length TFs, as exemplified by DLX5, allowing selection against biologically relevant full-length, multidomain proteins without immobilization or tags. TBS continues to function exclusively by selecting for the disruption of protein–DNA binding, ensuring mechanistic precision. Using this optimized TBS system, we successfully screened an 11.3-million-member peptide library to identify a potent antagonist of ATF2-CRE DNA binding within three months. This next generation TBS platform significantly improves screening efficiency and selection pressure while maintaining high biological relevance, providing a versatile and scalable tool for discovering functional peptide inhibitors of protein–DNA interactions with therapeutic potential.

## Graphical abstract

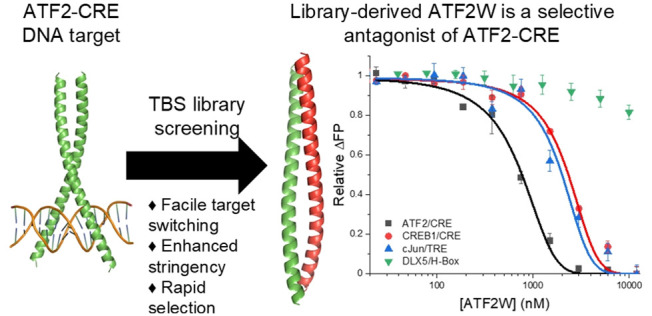

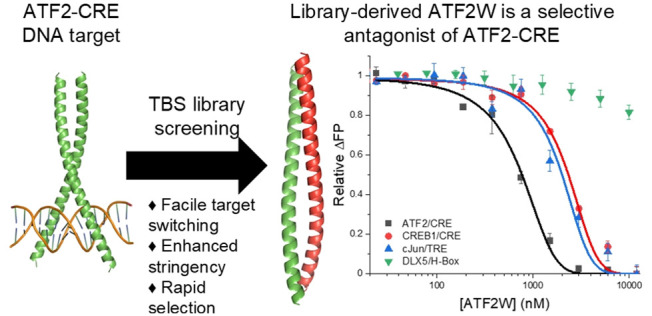

## Introduction

Peptides represent a highly promising class of therapeutic molecules in drug discovery that can bridge the gap between small molecules and biologics, combining favorable attributes of both.^1–5^ This unique positioning enables peptides to engage challenging drug targets that have been historically deemed “undruggable”. Advances in high-throughput genetically encoded peptide library screening technologies have further accelerated their discovery.^6–8^ These platforms operate by generating vast numbers of peptides which are either covalently linked or encapsulated with their corresponding nucleotide sequence. Peptides are then screened for their ability to bind or antagonize a target protein, with hit sequences rapidly identified via DNA sequencing.

The Transcription Block Survival (TBS) assay is a genetically encoded peptide library screening platform that selects for peptide antagonists of protein–DNA interactions in live bacterial cells.^7,9,10^ Unlike selection assays such as mRNA display or phage display, TBS produces a positive read-out only when a peptide functionally antagonizes its target by specifically disrupting DNA binding. Another bacterial system, FRep, has been developed to monitor protein–DNA binding *in vivo* through a related transcription truncation mechanism.^11^ However, TBS uniquely provides a simple and direct survival-based readout of TF antagonism of coexpressed peptides. The TBS assay exploits the essential role of dihydrofolate reductase (DHFR) in bacterial DNA synthesis and therefore cell growth. During screening, endogenous *E. coli* DHFR activity is selectively inhibited by trimethoprim (TMP), rendering growth is dependent on plasmid-encoded, TMP-resistant mouse DHFR (mDHFR). Target protein DNA-binding sites are inserted into the coding region of the mDHFR. When the target is expressed, its binding to these sites sterically blocks mDHFR transcription, preventing expression and thereby inhibiting cell growth. Plasmids encoding highly diverse peptide libraries are then transformed into TBS assay cells. Peptides that disrupt the target protein–DNA interaction relieve the transcription block, restoring mDHFR expression to promote cell growth. This creates a molecular dial, where peptide potency in inhibiting the protein–DNA interaction correlates directly with bacterial growth rate, enabling competition selection of the most effective antagonists.

Critically, screening peptides in a cellular context enables selection for additional drug-like properties such as target specificity, solubility, tolerability, and biostability, in parallel to functional antagonism. This built-in filtering process reduces time and resources spent on candidates with poor subsequent therapeutic potential. Moreover, TBS can screen millions of peptides in under a liter of media, making it significantly more sustainable and cost-effective than equivalent synthetic library approaches. TBS has already produced effective inhibitors of the oncogenic transcription factor (TF) cJun, which reduced cJun-driven melanoma cell viability.^10^ This demonstrated that hits identified by TBS retain efficacy in mammalian systems.

Although TBS has successfully identified inhibitors of cJun,^7,10^ BZLF1 ^9^ and other TFs (unpublished), a major limitation of the current protocol is the time and effort required to re-engineer the mDHFR reporter gene for each new target, as it must be modified to include the specific sequence recognized by the protein of interest. Since the target consensus sequence is inserted into the coding region of mDHFR, considerable time and effort is required to design variants that introduce sufficient binding sites while preserving function (i.e., by silent or conservative mutations) to produce the desired transcriptional block upon target protein expression. To address this limitation, we report a streamlined and optimized strategy for rapid target switching in TBS. We validated this optimized TBS system using cyclic AMP-responsive element-binding protein 1 (CREB1),^12,13^ activating transcription factor 2 (ATF2, also CREB2)^14–16^ and distal-less homeobox 5 (DLX5)^17–19^ targets, demonstrating effective transcriptional block and growth restoration upon peptide antagonism. Further, we report on the utilization of this enhanced TBS system to generate an effective peptide inhibitor of ATF2-DNA binding.

## Results and Discussion

This work was initiated following an unsuccessful attempt to develop a TBS system for selecting antagonists of cyclic AMP-responsive element (CRE)-binding TFs. As in previous designs, we first identified sites within the mDHFR gene that could be silently mutated to incorporate CRE sites. A second search targeted regions amenable to conservative amino acids substitutions. (i.e., similar side chain properties). These were limited to surface exposed amino acids to introduce additional CRE sites. Although this yielded an mDHFR variant containing five CRE sites in the coding region, the transcriptional block was insufficient, resulting in low assay stringency (Figure S1). While further optimization may have resolved this, the additional time and complexity required to adapt the reporter protein for each new target highlights a key limitation and is a barrier to the broader applicability and scalability of the TBS platform.

To improve the efficacy and versatility of TBS, we developed a modified assay design in which target DNA binding sites were inserted into the promoter and 5′ untranslated region (UTR) of the WT-mDHFR gene, rather than mutating its coding sequence (Figure 1). This region can be readily modified using standard cloning techniques to insert target sequences between *XhoI* and *SphI* restriction sites. As a proof of concept, we constructed two pES300d plasmids containing either five or nine CRE sites (Figures S2 and S3) to assess the number of binding sites required to instigate a robust transcriptional block. Notably, CRE sites were introduced at the positions of the two lac operator sites in the plasmid. Although mutations to the lac operator may affect repressor function, this is not required during TBS screening, as mDHFR expression is controlled by the transcription block imposed by target protein–DNA binding. In the 5xCRE WT mDHFR construct, three additional sites were introduced into the 5′ UTR. For the 9xCRE WT mDHFR construct, the 5′ UTR was extended to accommodate six CRE sites, with a ninth site placed in the 5′ region of the T5 promoter.

**Figure 1:**
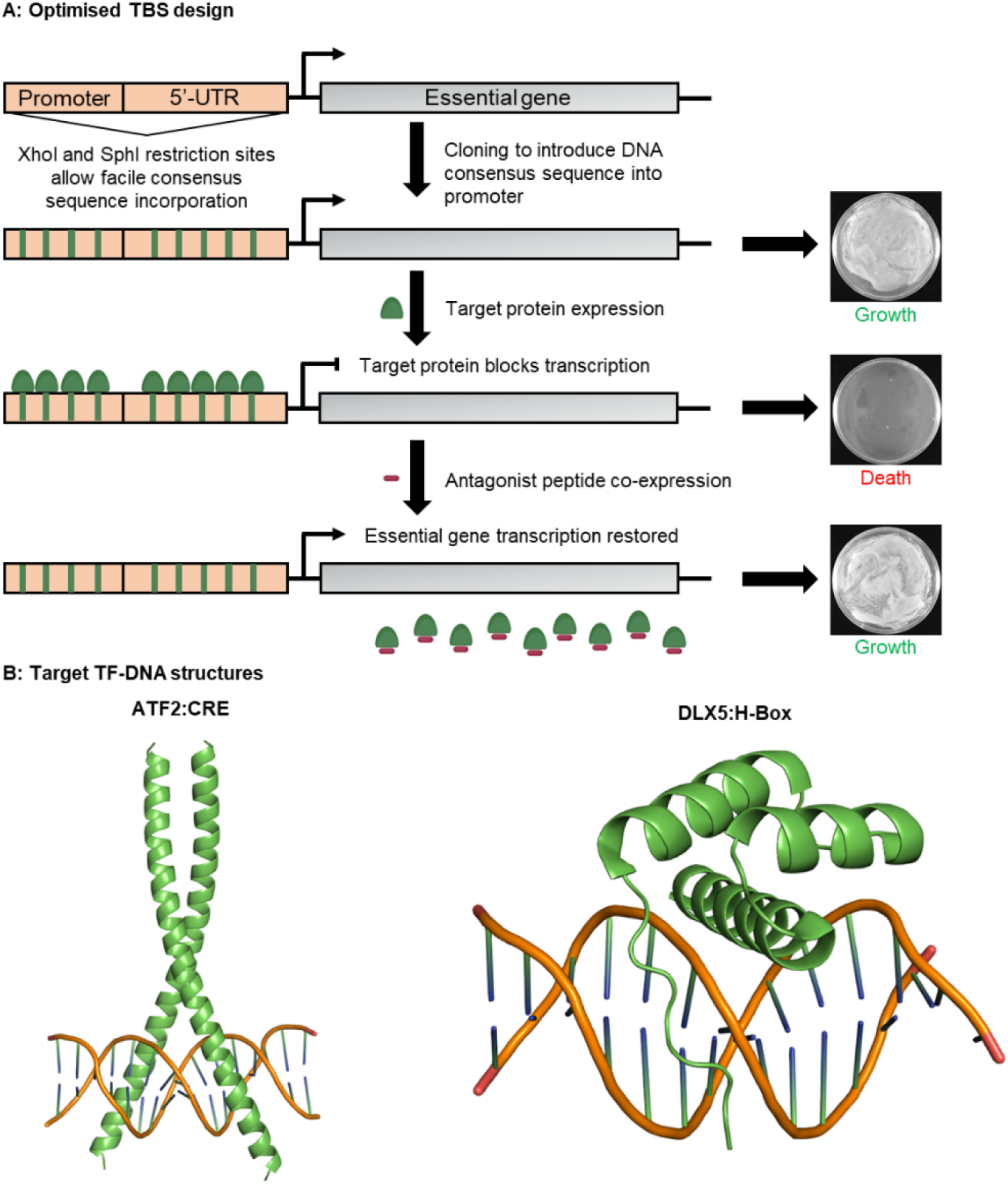
Streamlined TBS methodology facilitates rapid target switching with enhanced selection stringency against new oncogenic targets. (A) TBS assay design schematic illustrating the facile incorporation of target consensus sites into the 5′-promoter/UTR of the mDHFR gene. Upon target binding to these sites the essential gene transcription is blocked, abrogating bacterial growth. Introduction of an effective functional antagonist removes the transcription block and restores bacterial growth, allowing for a direct correlation between peptide antagonist efficacy and growth rate. (B) Alphafold prediction of the ATF2 bZIP domain homodimer bound to CRE DNA (pTM score: 0.85, iPTM score: 0.84, plDDT > 90) and the crystal structure of the DLX5 homeodomain bound to H-Box DNA (PDB ID: 4RDU).

To validate the new TBS constructs, six BL21-Gold *E. coli* control strains were generated. Empty plasmids (lacking gene of interest) were used to control for replication and antibiotic stresses (Table 1): Control strains 1 and 4 contained mDHFR gene reporter plasmids for 5xCRE and 9xCRE respectively, and two empty plasmids; control strains 2 and 5 contained reporter plasmid, target plasmid encoding the CREB1 basic-leucine zipper (bZIP) domain and one empty plasmid; control strains 3 and 6 contained reporter plasmid, target plasmid and the antagonist plasmid encoding A-CREB (peptide inhibitor of CREB1 comprising a rationally designed acidic extension appended to the N-terminus of the CREB1 LZ).^20^

**Table 1:** BL21-Gold *E. coli* Control Strains for TBS Validation^a^

TBS Control Strain	pES300d	pES230d	pQE80
1	5xCRE mDHFR	Empty	Empty
2	5xCRE mDHFR	CREB1 bZIP	Empty
3	5xCRE mDHFR	CREB1 bZIP	A-CREB
4	9xCRE mDHFR	Empty	Empty
5	9xCRE mDHFR	CREB1 bZIP	Empty
6	9xCRE mDHFR	CREB1 bZIP	A-CREB
7	9xCRE mDHFR	ATF2 bZIP	Empty
8	9xDLX mDHFR	Empty	Empty
9	9xDLX mDHFR	DLX5 HD	Empty
10	9xDLX mDHFR	DLX5 FL	Empty

a Each control strain contains three pQE16 plasmid derivatives to facilitate reliable comparison of growth rates , with empty plasmids utilized where no expression is required.

When plated on selective agar, robust growth of positive control strains 1 and 4 confirmed that mDHFR was expressed and conferred resistance to TMP. In contrast, strains 2 and 5, which expressed the CREB1 bZIP domain, exhibited markedly reduced growth, indicating effective transcription block resulting from the CREB1 bZIP binding to CRE sites in the mDHFR reporter construct. Growth was restored in strains 3 and 6 upon expression A-CREB, a known antagonist of the CREB1-CRE interaction,^20^ demonstrating that inhibition of target DNA binding relieved the transcriptional block, reactivating mDHFR expression, reinstating TMP resistance, leading to *E. coli* growth.

Strains carrying the reporter plasmid with 9xCRE sites showed a greater reduction in growth than those with 5xCRE sites (strain 5 vs strain 2), indicating that increasing the number of CRE sites enhances the degree of transcriptional shutdown. Since this enhanced transcription block yielded greater stringency (i.e reduced cell viability), all subsequent investigations used the 9xCRE reporter construct with CRE sites positioned in the 5′-promoter/UTR region of the mDHFR gene. Similar transcriptional repression and growth inhibition were observed when CREB1 was replaced by the alternative CRE-binding TF, ATF2 (Table 1, strains 6 and 7; Figures 1B and 2), demonstrating the platforms broader applicability to other CRE-binding protein targets.

**Figure 2:**
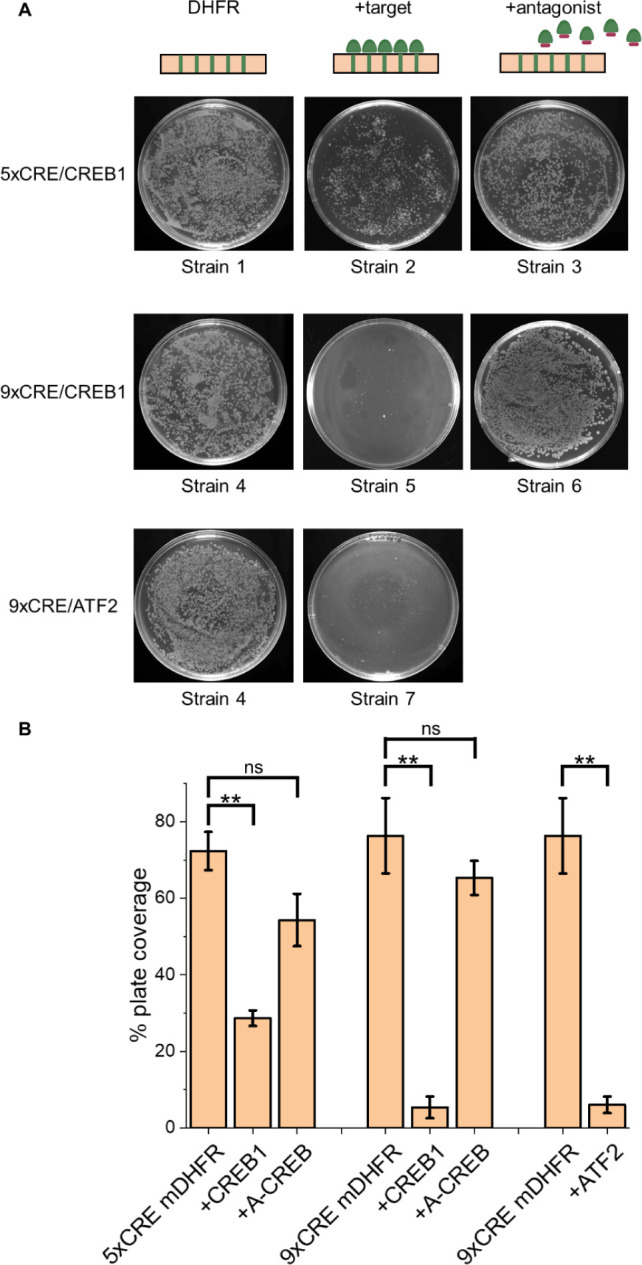
Introduction of CRE DNA sites into the 5′-promoter/UTR region of a mDHFR plasmid facilitates effective transcription block. (A) Equal numbers of cells from the control strains (Table 1) were plated on selective M9 agar (8 μM TMP, 1 mM IPTG) and incubated before imaging. Images are representative of triplicate experiments. (B) Bar charts show % plate coverage for three independent experiments with error bars shown as one standard deviation. Select *p* values from a *t* test are indicated (**p ≤ 0.01; ns = not significant). A-CREB is a previously developed CREB1 antagonist, utilized here as a positive control showing bacterial growth restoration.

To highlight the platforms versatility for TFs with distinct DNA recognition motifs, we next constructed a 9xhomeobox (H-Box) system to identify inhibitors of homeodomain-containing TFs, including the DLX (Figure 1C), pre-B cell leukemia transcription factor (PBX) and orthodenticle homeobox protein (OTX) families. As with the CRE-based design, nine H-box consensus sequences were inserted into the 5′-promoter/UTR region of the mDHFR reporter gene (Figure S4).

The positive control, strain 8, transformed with the H-box containing reporter plasmid and empty target and antagonist plasmids was able to grow in TBS assay conditions, at all IPTG concentrations and incubation temperatures tested, which indicated expression of the mDHFR reporter gene to replace the TMP-inhibited endogenous DHFR activity (Figure 3). Consistent with the other TBS systems, expression of DLX5 homeodomain (HD) or full-length DLX5 (FL) introduced a transcriptional block, resulting in reduced colony formation (Figure 3, strains 8 vs 9 and 10). Notably, the growth inhibition observed with the DLX5-HD (strain 9) was less pronounced than in the corresponding CRE system, likely reflecting differences in binding affinity and interaction mode of the respective protein–DNA interactions.

**Figure 3:**
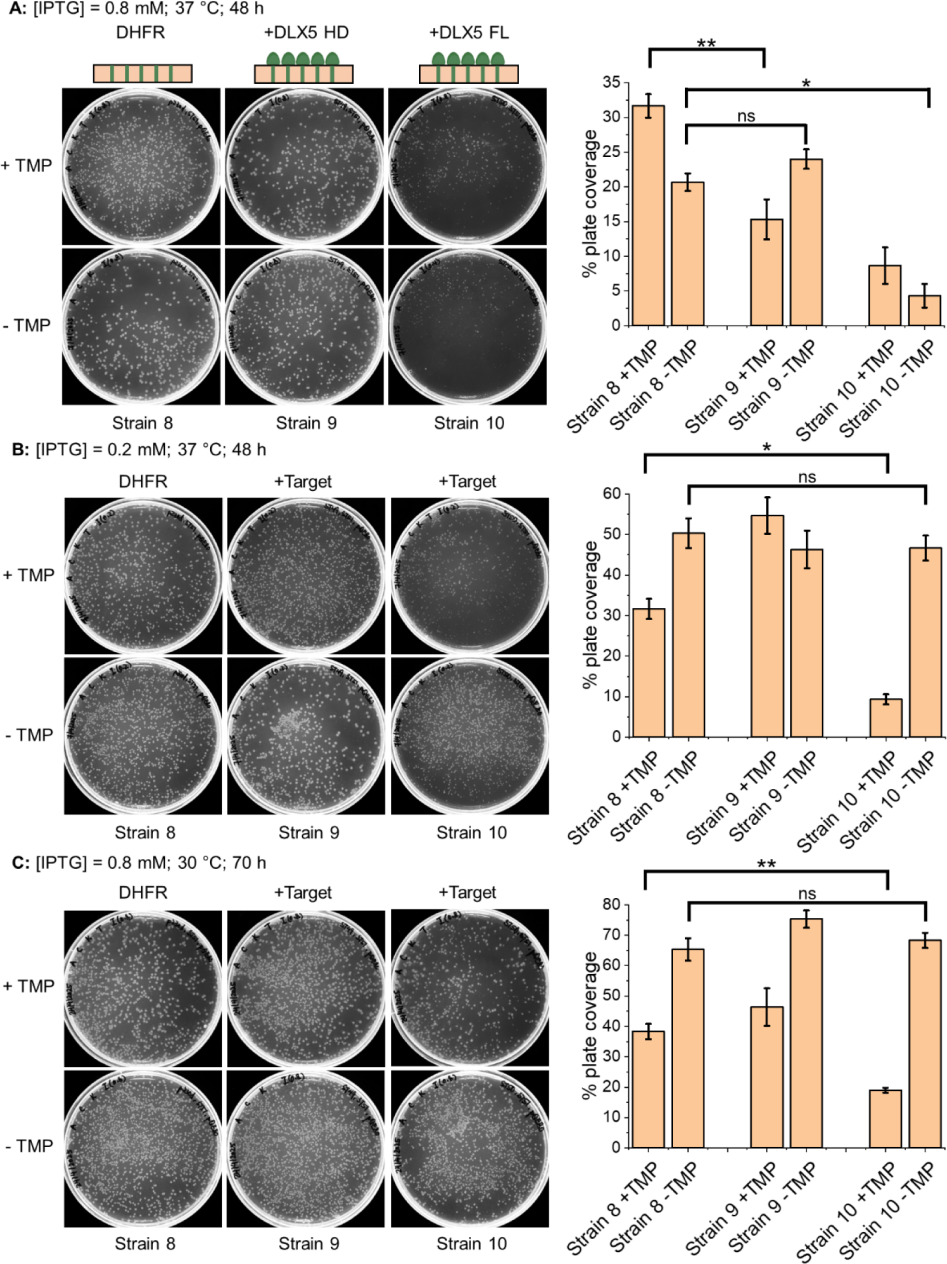
Introduction of H-box DNA sites into the 5′-promoter/UTR region of a mDHFR plasmid enables transcription block by both DLX5 full length and homeodomain. Equal numbers of cells from the control strains (Table 1) were plated on selective M9 agar (0 or 8 μM TMP, 0.8 or 0.2 mM IPTG) and incubated (30 or 37 °C) before imaging. Bar charts show % plate coverage for three independent experiments with error bars shown as one standard deviation. Select *p* values from a *t* test are indicated (*p ≤ 0.05; **p ≤ 0.01; ns = not significant).

Although expression of DLX5 FL (strain 10) produced more significant growth inhibition than the DLX5 HD construct (strain 9), reduced growth was also observed in the absence of TMP, suggesting that high-level expression with 0.8 mM IPTG caused cellular toxicity (Figure 3A). This was mitigated by either reducing IPTG concentration to 0.2 mM (Figure 3B) or lowering the incubation temperature to 30 °C (Figure 3C). Under these optimized conditions, DLX5 FL produced a robust transcriptional block and more effectively suppressed *E. coli* growth than DLX5 HD, indicating that future TBS screening will be more effective using the FL target protein.

This validates the new TBS design for the introduction of a transcriptional block and for the ability of antagonist peptides to restore bacterial growth in the CRE/CREB systems. We next constructed an 11.3-million-member peptide library to identify antagonists of ATF2, representing the first such effort to target this TF. This library design includes an N-terminal acidic domain, developed by Vinson and coworkers to engage bZIP DNA-binding domains (DBDs), followed by a semirandomized LZ region. Canonical coiled coil heptad repeat **e** and **g** positions were semirandomized to include glu, gln or lys options, with two exceptions to increase library diversity: **e7** (ile, asn, asp or val) and **g8 (**leu, gln, glu or val). The **a** positions were randomized to select from leu, ile or val, to optimize hydrophobic packing in the coiled coil core, except **a6** which also included asn or asp to investigate potential core polar interactions. The **d** positions were fixed as leu to promote formation of a desired parallel, dimeric coiled coil. Noninterfacial **b**, **c** and **f** positions were also fixed. The library was generated by whole-plasmid PCR and transformed in *E. coli*, yielding 55.5 million colony-forming units, providing a 99.3% probability of full library coverage.

The peptide library was transformed into BL21 Gold TBS *E. coli* containing the p300 9xCRE-mDHFR reporter and p230 ATF2 bZIP expression plasmids. A lawn of colonies formed on selection plates, and dilution plating confirmed a 99.1% probability of full library coverage. Following nine rounds of selection, a single dominant hit emerged: ATF2W (Figures 4 and S5). The ATF2W sequence was observed by individual colony sequencing during every passage and was the only sequence observed after passage nine. *E. coli* expressing ATF2W monopolized the culture due to the growth advantage imparted by this sequence, through inhibition of the target interaction. Both ATF2 and ATF2W peptides were synthesized via standard Fmoc-protected solid phase peptide synthesis and purified by RP-HPLC for subsequent biophysical characterization (Figures S6 and S7).

**Figure 4:**
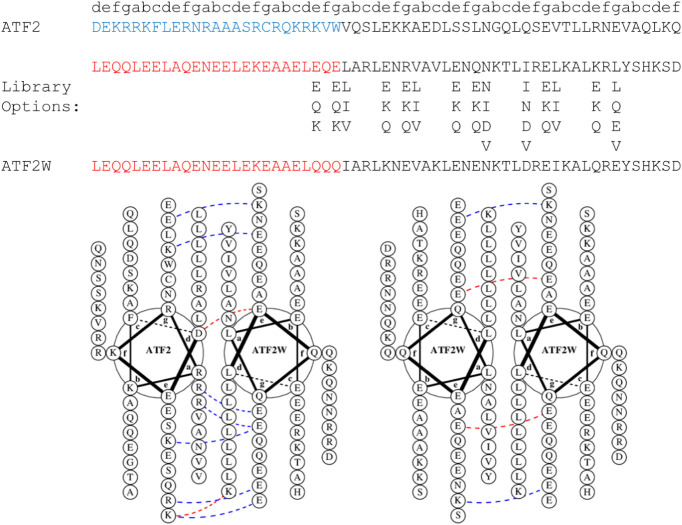
Target ATF2, antagonist library design and TBS assay winner peptide ATF2W sequences. Helical wheel diagrams illustrate the desired ATF2-ATF2W interaction and undesired ATF2W-ATF2W homodimer interaction.

Binding between ATF2 and ATF2W was first characterized using circular dichroism spectroscopy to detect changes in global secondary structure upon complex formation. CD spectra were collected for each peptide individually (10 μM) and for a 1:1 mixture (5 μM each), with peptide concentration held constant to account for the concentration dependence of dimer helicity. An average spectrum of the individual components was determined, as this represents the expected spectrum in the case of no interaction. All sample spectra displayed a variable minimum at 222 nm, indicative of α-helical content. Notably, a 32% increase in helicity was observed upon mixing, relative to the average, indicating coiled coil heterodimer formation (Figure 5A). The 222/208 ratio of the heterodimer spectrum is 0.8, with values of ≥ 1 typically observed for classical coiled coils.^21^ This likely indicates that the N-terminal acidic extension of ATF2W does not form a canonical coiled coil interaction with the DBD, though it has been shown to enhance binding in the original work by Vinson et al.^20^ To prevent disulfide formation ATF2 samples were incubated with 10 mM TCEP.HCl for 1 h incubation, which also produced significant noise in the spectra. To further investigate the interaction, thermal denaturation was monitored at 222 nm between 1 and 95 °C. The heterodimeric ATF2/ATF2W sample displayed a *T*_*m*_ of 55 °C, representing a *ΔT*_*m*_ of 18 °C relative to the ATF2W alone which displayed a *T*_*m*_ of 37 °C (Figure 5B). This significant enhancement in helicity and thermal stability supports the formation of a helicity-inducing coiled coil binding interaction between ATF2 and ATF2W. Finally, isothermal titration calorimetry (ITC) was used to determine the thermodynamic parameters of the ATF2 (5 μM)-ATF2W (50 μM) binding interaction, which indicated a *K*_*D*_ = 114 ± 21 nM (Figure 5C, *N* = 0.99 ± 0.01, *ΔH* = −79 ± 3 kJ mol^–1^, *-TΔS* = 40 ± 4 kJ mol^–1^).

**Figure 5:**
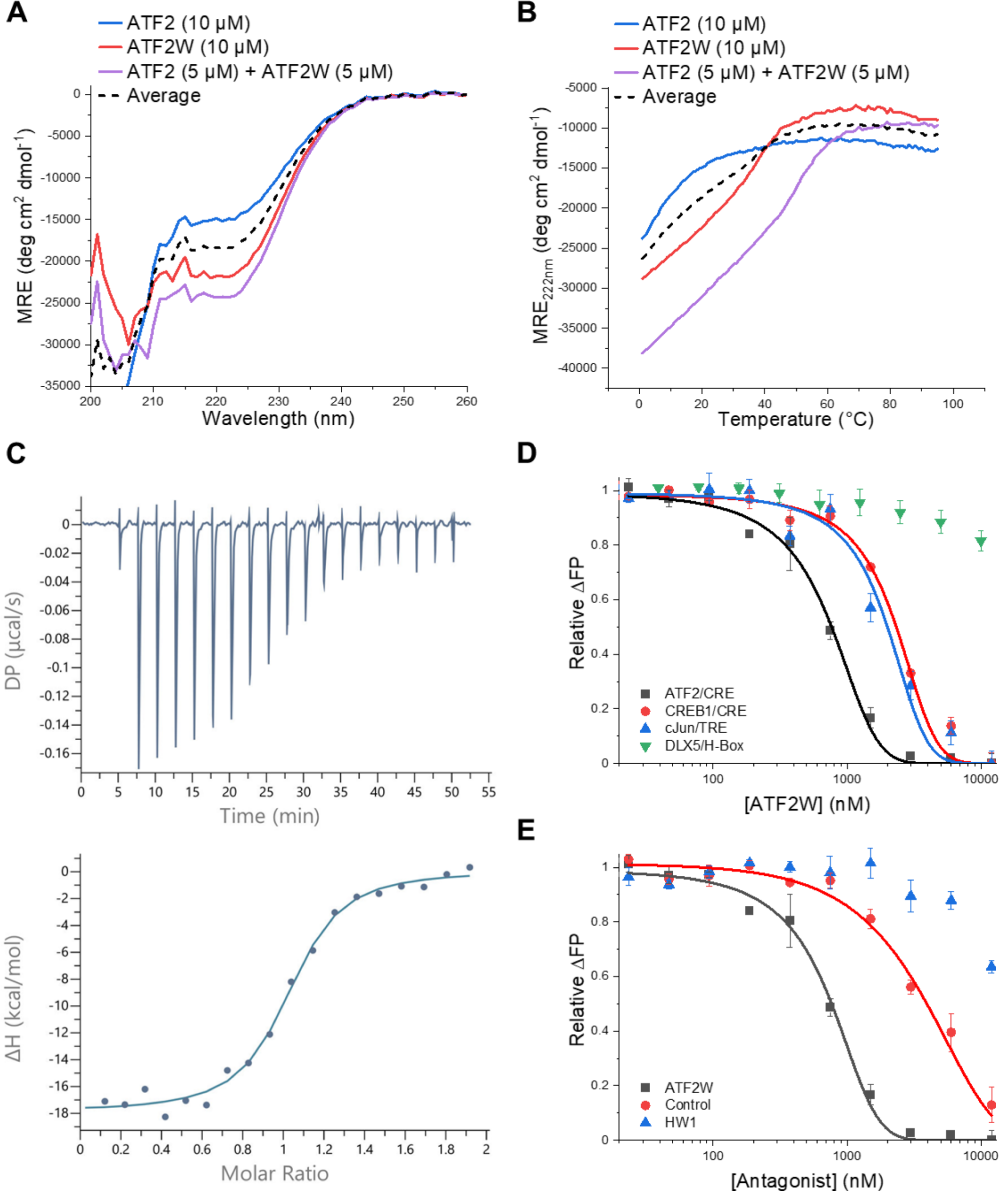
Biophysical characterization of the ATF2-ATF2W interaction. (A) CD spectra of ATF2 and ATF2W indicating an increase in helicity upon mixture of the two components, indicative of the formation of a heterodimeric coiled coil. (B) CD thermal denaturation experiment indicates a *T*_m_ shift upon mixture of ATF2/ATF2W, supporting the formation of a stable heterodimer. The “average” CD trace in A and B represents an averaged value of the two unbound components which would be the trace for no interaction. (C) ITC data for ATF2W (50 μM) titrated into ATF2 (5 μM) at 20 °C. The raw power compensation plot is shown in the upper graph and the integrated data points and single site model fitted line are shown in the lower graph. (D) FP antagonism assay shows that ATF2W is capable of inhibiting the ATF2-CRE DNA interaction, at the top concentrations reducing the FP value to that of free DNA. ATF2W is selective for ATF2, with a ∼ 3-fold reduction in antagonism for CREB1/CRE and cJun/TRE despite high structural and sequence homology. Minimal antagonism of the DLX5/H-Box complex was observed. (E) FP assay illustrates that ATF2W produces more effective ATF2-CRE antagonism than a nonselected library member control and a previous TBS-derived cJun inhibitor peptide. This indicates that less active antagonists are effectively deselected during TBS screening.

Fluorescence polarization (FP) was next utilized to confirm that ATF2W binding induces the intended antagonism of the ATF2-CRE interaction. First, a FP binding curve was generated by equilibrating ATF2 (12 nM-10 μM) with 10 nM FAM-labeled CRE DNA, yielding a *K*_*D*_ of 277 ± 19 nM (Figure S8). To assess ATF2W antagonism, 625 nM ATF2, which corresponded to 80% of the maximum DNA binding, was incubated with CRE DNA (10 nM) and varied concentrations of ATF2W (29 nM-12 μM). A dose-dependent decrease in FP signal was observed, indicating DNA release upon ATF2W binding, with an *IC*_50_ of 667 ± 71 nM (Figures 5D and S9–S14). Selectivity testing revealed that ATF2W antagonized related CREB1-CRE and cJun-TRE complexes, which are structurally identical bZIP DBDs and share 38% and 44% sequence identity (60% and 66% sequence similarity) to ATF2, with *IC*_50_ values of 2365 ± 59 and 1851 ± 213 nM, respectively. This represents a ∼ 3-fold selectivity for ATF2. Further, minimal antagonism was observed of the DLX5/H-Box interaction over the concentration range tested. FP confirmed that ATF2W does not interact with CRE DNA (Figure S15), supporting its role as a protein–protein interaction antagonist rather than a DNA binder. To demonstrate the utility of TBS for selecting enhanced antagonists, we synthesized and assessed a deselected library member sharing 85% sequence identity with ATF2W (Figures S16 and S17). This control peptide inhibited the target interaction with an IC_50_ of 3688 ± 110 nM, representing a 5.5-fold reduction in activity compared with the winning sequence (Figure 5E). Additionally, a previous TBS-derived cJun inhibitor, HW1,^7^ showed markedly lower efficacy against ATF2/CRE (Figures 5E and S18). As the peptide library was designed using established coiled coil interaction rules, it was expected that many sequences would bind the target. Nonetheless, these results show that TBS screening can deselect highly homologous but less effective antagonists.

## Conclusions

The TBS platform is a highly powerful intracellular peptide screening system for identifying peptide antagonists of TFs. It has been successfully employed to generate effective peptides that disrupt TF-DNA interactions, leading to effective inhibition of target function.^7,9,10^ However, earlier iterations of TBS required laborious redesign of the mDHFR reporter gene to incorporate new DNA recognition sequences for each TF, limiting the platform’s adaptability and broader application to other TF targets.

Our studies represent a significant advancement of the TBS screening platform by overcoming previous limitations through strategic redesign of the reporter plasmid to enable rapid and flexible target switching. In this improved system, TF binding sites were relocated from the translated region of the mDHFR gene into the 5′ UTR and promoter region. This means that the 5′ UTR and promoter region is the only region that requires editing ahead of TBS screening. This design eliminates the need to redesign the coding sequence for each iteration, where changes could compromise reporter function and assay integrity. Additionally, the use of WT-mDHFR in place of the previously modified TRE-mDHFR variant results in increased enzymatic activity,^7^ promoting faster bacterial growth and thereby accelerating the TBS screening process.

The current study investigated the use of five or nine DNA recognition sites to incur the required transcription block in a CREB1/CRE DNA system. Reporter plasmids containing nine binding sites in the 5′ UTR and promoter were proven to generate highly effective transcription blocks for both CRE- and H-Box mDHFR. Further optimization of number and placement of DNA binding sites could be performed to balance growth rate and stringency during selection. CREB1 and ATF2 were employed to evaluate the utility of the CRE-mDHFR reporter plasmid against different CRE-binding TFs. Reduction in *E. coli* growth, indicating effective transcription blocks were observed in both instances. As proof-of-concept, an 11.3-million-member peptide library was screened against ATF2 by TBS using the CRE-mDHFR reporter plasmid. This resulted in the selection of a single peptide sequence, ATF2W, as the first *de novo* ATF2 antagonist, capable of binding to ATF2 and sequestering it from its cognate CRE DNA. This validates the development of this redesigned system, where previously we were unable to produce antagonists of CRE-binding TFs due to a lack of stringency in the assay. We have also demonstrated that although a highly homologous, nonselected library member was capable of antagonizing the target interaction, the TBS assay was able to differentiate between the 5.5-fold difference in their activities to select an optimal sequence. While selection in the TBS system does not ensure cancer cell efficacy (as with any screening approach), we have previously shown that TBS-derived peptides reduce viability in relevant cancer cell lines,^10^ and the built-in selection for specificity, solubility, tolerability, and biostability in the system increases the likelihood of beneficial pharmacokinetic and pharmacodynamic properties in hit sequences. Other bacterial assays such as FRep have demonstrated *intracellular* reporting of protein–DNA interactions using FRET,^11^ but these signals rely on expression levels, variable linker length and architecture, and can be perturbed if peptides bind CFP or YFP. While the FRep system offers greater sensitivity for detecting single binding events via changes in fluorescence, it remains unclear whether TBS can currently resolve such single-site interactions. By contrast, TBS provides a clean growth/no-growth functional peptide selection readout, avoiding fluorescence-based artifacts and reducing the risk of false positives when screening for antagonists.

The α-helical coiled coil motif poses inherent challenges for achieving target selectivity. Its interactions are largely driven by core hydrophobic packing, meaning that any sequence containing a leucine zipper motif is likely to bind a target bZIP to some extent. In the case of the CREB1 and cJun bZIP domains, the canonical heptad binding surfaces (positions ***a***, ***d***, **e**, and ***g***) share 73% and 70% sequence similarity, respectively, with those of the target ATF2. Despite this high homology, the ATF2W peptide selectively antagonized ATF2/CRE binding compared with CREB1/CRE, cJun/TRE, and DLX5/H-Box, achieving a 3-fold selectivity for ATF2 over CREB1 and cJun. While this demonstrates that selection for enhanced ATF2 antagonism can yield a measurable degree of selectivity, the current TBS platform was not optimized to discriminate against other bZIP family members. Future iterations could incorporate competitor proteins and negative design strategies to drive the discovery of highly selective peptides. Importantly, for therapeutic purposes, even a 3-fold selectivity over closely related off-targets in biophysical assays may be sufficient to achieve an acceptable therapeutic window, especially when combined with in vivo expression and localization differences.

We have expanded the TBS platform beyond bZIP domain inhibition, by producing a system for screening against HD-containing DLX5. Interestingly, the HD alone only produced a subtle reduction in *E. coli* growth, indicative of insufficient DNA binding to produce the desired steric transcriptional block. With optimization to overcome the toxicity related to the expression of DLX5 FL, more significant transcription block-induced reduction in *E. coli* growth was observed with the full-length TF. The more effective transcription block exhibited by full-length protein suggests that other regions/domains of DLX5 beyond the HD offer significant contribution toward recognition and binding to DNA. Liu et al. demonstrated that distinct intrinsically disordered regions of HD-containing *Drosophila* ultrabithorax protein either enhanced or inhibited DNA binding.^22^ While this study showed that the ultrabithorax HD bound DNA more efficiently than the full-length protein, it follows that with other proteins such as DLX5 the overall contributions of the full-length protein will lead to increased full-length protein–DNA binding, compared to HD-alone, as our study has suggested.

Critically, this H-box system using a full-length TF containing intrinsically disordered regions demonstrated a key advantage of the TBS assay. Many peptide library screening approaches require purified and immobilized target proteins. Purification of large intrinsically disordered proteins is typically challenging and sometimes impossible, and immobilizing can obscure potential binding sites. Intracellular methods such as TBS overcome this limitation by enabling *in situ* peptide library screening during recombinant expression within *E. coli*, facilitating screening against any protein target without the need for purification, or the addition of fusions/tags. Future work will utilize these systems to generate CREB1 and DLX5 antagonists.

In summary, the optimized TBS platform, enabled by a redesigned reporter system, allows for rapid and efficient target switching, and was successfully applied to three TFs across two distinct cognate DNA recognition sequences. The updated plasmid design not only simplified adaptation to new targets but also enhanced selection pressure and increased growth rates during screening, significantly improving throughput. Collectively, these improvements enabled the identification of an effective antagonist of ATF2-CRE DNA binding within just three months (from cloning and TBS validation, through library construction and screening, to peptide synthesis and biophysical characterization), demonstrating the platform’s powerful utility for intracellular peptide discovery.

## Supplementary Material



## Notes

Further detailed information relating to materials and methods utilized in this work is provided (PDF)
